# Cross-national comparison of psychosocial well-being and diabetes outcomes in adults with type 1 diabetes during the COVID-19 pandemic in US, Brazil, and Iran

**DOI:** 10.1186/s13098-021-00681-0

**Published:** 2021-06-11

**Authors:** Samereh Abdoli, Monica S. V. M. Silveira, Mehri Doosti-Irani, Paulo Fanti, Katherine Miller-Bains, Elizabeth João Pavin, Edimariz Buin Cardoso, Leila Rafiee Vardanjani, Kobra Noorjan, Danielle Hessler

**Affiliations:** 1 College of Nursing, University of Tennessee, 1200 Volunteer Blvd Rm 155, Knoxville, TN 37996 USA; 2 Faculty of Medical Sciences, University of Campinas, Rua Tessália Vieira de Camargo, 126. Cidade Universitária Zeferino Vaz, Campinas, São Paulo, 13083-887 Brazil; 3 School of Nursing and Midwifery, Shahrekourd University of Medical Sciences, Shahrekord, Iran; 4 Assessment and Evaluation, Oak Ridge Associated Universities, Oak Ridge, TN 37831 USA; 5 Endocrinology Division, Department of Internal Medicine, Faculty of Medical Sciences, University of Campinas, Rua Tessália Vieira de Camargo, 126. Cidade Universitária Zeferino Vaz, Campinas, São Paulo, 13083-887 Brazil; 6 Clinical Psychologist, Faculty of Medical Sciences, University of Campinas, Rua Tessália Vieira de Camargo, 126. Cidade Universitária Zeferino Vaz, Campinas, São Paulo, 13083-887 Brazil; 7 Department of Family and Community Medicine, University of California, 500 Parnassus Avenue, San Francisco, CA 94117 USA

**Keywords:** COVID-19, Burnout, Distress, Depression, Diabetes

## Abstract

**Background:**

The COVID-19 pandemic is a global public health emergency, which presents wide-ranging negative impacts on individuals with diabetes. To examine psychosocial well-being and diabetes outcomes in individuals with type 1 diabetes during the COVID-19 pandemic, and investigate how these factors vary in different countries.

**Methods:**

Between April and June 2020 we employed a cross national comparative research study in the United States (US), Brazil, and Iran to collect data from 1788 adults with type 1 diabetes using web-based survey. Study participants answered questions relevant to diabetes distress, diabetes burnout, depressive symptoms, COVID-19 related changes, and socio-demographic characteristics. They also reported their last Hemoglobin A1c (HbA1c) and daily Time-in-Range (TiR) blood glucose. We analyzed data using comparative tests (Chi-square, Kruskal–Wallis and McNemar test), logistic and linear regression adjusted for fixed effects.

**Results:**

There were significant changes prior and during the pandemic regarding access to diabetes care, diabetes supplies and medications, healthy food and safe places to exercise in all countries (*p* < 0.05). Participants in Iran experienced higher levels of diabetes distress (57.1%), diabetes burnout (50%), and depressive symptoms (60.9%), followed by Brazil and US (*p* < 0.0001). US participants reported better glycemic control (HbA1c = 6.97%, T1R = 69.64%) compared to Brazil (HbA1c = 7.94%, T1R = 51.95%) and Iran (HbA1c = 7.47%, T1R = 51.53%) (*p* < 0.0001). There were also significant relationships between psychosocial well-being, diabetes outcomes, socio-demographic data, and COVID-19 related challenges in overall sample (*p* < 0.05).

**Conclusions:**

Regardless of differences among US, Brazil, and Iran, our findings revealed that different countries may experience similar challenges related to the COVID-19 pandemic which can impact negatively diabetes outcomes and psychosocial well-being in individuals with type 1 diabetes. Countries need to consider modifiable variables associated with poor diabetes outcomes and sub optimal psychosocial well-being and target vulnerable population using significant socio-demographic variables.

**Supplementary Information:**

The online version contains supplementary material available at 10.1186/s13098-021-00681-0.

## Novelty statement

- This study for the first time examine diabetes distress, diabetes burnout, and depressive symptoms in a cross national sample of individuals with type 1 diabetes.
- This study highlights significance differences in access to diabetes care, medications and supplies, healthy food and safe places to exercise during the COVID-19 pandemic regardless the country of origin.

## Background

Coronavirus disease 2019 (COVID-19), was first reported in Wuhan, China, reaching a pandemic level in a few months [1] with a mortality rate ranged from 0 to 14.6% [2]. During the pandemic, US, Brazil, and Iran were significantly affected by the pandemic. Until March 8, 2021, these countries ranked highest in numbers of COVID-19 positive cases among countries in North America, South America, and Middle East with a total confirmed cases of 32.69 million for US, 15.15 million for Brazil and 2.64 million for Iran. The daily confirmed new cases for US, Brazil and Iran were 42,012; 59,986; and 17.787 and deaths confirmed numbers in these countries were respectively 581,516 (US), 421,316 (Brazil), and 74,524 (Iran) [3].

The COVID-19 pandemic is a global public health emergency, which presents profound and wide-ranging negative impacts on vulnerable communities, including individuals living with diabetes [4]. The prevalence of diagnosed and undiagnosed diabetes estimates among the US population is 34.1 million adults aged 18 years or older, or 13.0% of all US adults [5]. In Brazil, diabetes is a significant public health concern with a prevalence of 16.8 million or 10.5% of all Brazilian adults [6]. Similarly, diabetes in Iran affects 11.4% of adult population [7]. Considering the high prevalence of diabetes in US, Brazil and Iran, the COVID-19 pandemic may represent an unprecedented challenge for individuals with diabetes in these countries. Despite the existing uncertainties, individuals with diabetes are categorized as “at risk” population [8]. Unique demands of diabetes care, particularly type 1 diabetes, in this population (i.e., consistent demand of monitoring of blood glucose, insulin dosing, meal planning) may be further amplify during the pandemic. There are evidence suggesting routine diabetes care for Type 1 diabetes may be significantly disrupted by the COVID-19 pandemic [9]. Access to insulin, medications, strips, and medical equipment has been hampered in many places [10]. Lack of access to healthy food, safe places to exercise, and financial stress has also intensified the negative impact of pandemic in individuals with type 1 diabetes [11]. During the pandemic, increased acute complications of diabetes (i.e., DKA) and delay in seeking medical support has also been reported in this population [12]. In addition, COVID-19 pandemic has forced many hospitals to implement significant changes in their care structure, and diabetes providers were relocated to care for COVID-19 infected individuals [10]. Disruption to routine diabetes care, social isolation, and quarantine is also associated with higher levels of stress, fear of becoming infected, and concerns related to availability of appropriate care in individuals with type 1 diabetes [13, 14]. This can contribute to worsening diabetes management, resulting in long-term diabetes complications and suboptimal diabetes outcomes [10]. These combined with disparities in socio-determinants of health may negatively impact their psychosocial well-being [15]. The need for both universal and targeted mitigation of COVID-19’s psychosocial impact is now rising globally. The World Health Organization (WHO) declared the need for a rapid assessment of the context and of culturally specific psychosocial well-being issues, needs and available resources as key activities of the response to COVID-19 [16]. The National Health Organization (NIH) encourages rapidly understanding of the critical psychosocial and behavioral aspects of the COVID-19 pandemic [17]. Research data are needed to include the voices and needs of the population in planning and emergency response to maintaining both physical and psychosocial well-being while reducing risk of being infected with COVID-19. The immediate research goals should be screening psychosocial well-being across vulnerable and high risk groups, including individuals with diabetes [18].

Diabetes distress, diabetes burnout, and depressive symptoms are common in individuals with diabetes [19–21], and are associated with difficulties in diabetes self-management, and sub-optimal glycemic control regardless of the pandemic [21–23]. Given this unique pandemic scenario and the lack of scientific evidence, the current study aims to 1: compare psychosocial well-being (i.e., diabetes distress, diabetes burnout, and depressive symptoms) and glycemic control (i.e., last self-reported HbA1c and daily TiR) among adults with type 1 diabetes experiencing the COVID-19 pandemic in US, Brazil, and Iran, and 2: examine which COVID-19 related changes and sociodemographic characteristics are related to psychosocial well-being and glycemic outcome in these countries. During study data collection.

By capturing countries with different socio-demographic information, it provides a broader spectrum of data to determine what factors affect the outcomes in different countries. To our knowledge this is the first cross-national analysis on psychosocial well-being and diabetes outcomes in individuals with diabetes during the pandemic which can inform future national health initiatives.

## Materials and methods

### Design

During the COVID-19 pandemic (April 1st–June 30th 2020), we conducted a cross-national comparative study using web-based survey to collect data from adults with type 1 diabetes in US, Brazil, and Iran. A cross-national comparative study compares the same concepts in two or more countries to make generalizations or gain a better understanding of the phenomena under study [24]. During the COVID-19 pandemic, this methodology offers the opportunity to improve the international understanding of psychosocial well-being and diabetes outcomes in individuals with diabetes. During the study time period, the number of daily new confirmed COVID-19 cases were 41,710.86 in US, 36,590.71 in Brazil, and 2527.43 in Iran. The number of daily new confirmed COVID-19 deaths per million in Iran (4.67) and US (1.58) were similar while Brazil experienced a higher number of death (4.67) at the same time period. All study activities were approved by the Institutional Review Board (IRB) at the University of Tennessee (UTK IRB #18-04540-XP) in US, the University of Campinas Ethics in Research Committee (CAAE # 30899220.7.0000.5404) in Brazil, and the Shahrekourd University of Medical Sciences, Ethics in Research Committee (IR SKUMS REC #1399.051) in Iran. We used the checklist for Reporting Results of Internet E-Surveys (CHERRIERS) which to ensure the quality of our web based survey report [25].

### Study sample and recruitment

Study flyers were displayed by social media, diabetes support groups, and diabetes clinics in US, Brazil, and Iran. In US, flyers were also distributed to potential participants by T1D Exchange registry. The survey was an open survey visible for each online visitor. The survey was a voluntary survey. Individuals aged 18 years or older, diagnosed with type 1 diabetes and interested to participate at the study had access to a link directing them to a landing page including study consent form, screening survey, followed by a survey battery if eligible to participate. To reduce sampling, measurement and data collection bias, we utilized the same inclusion criteria, data collection process, and measures across the different countries.

**Fig. 1:**
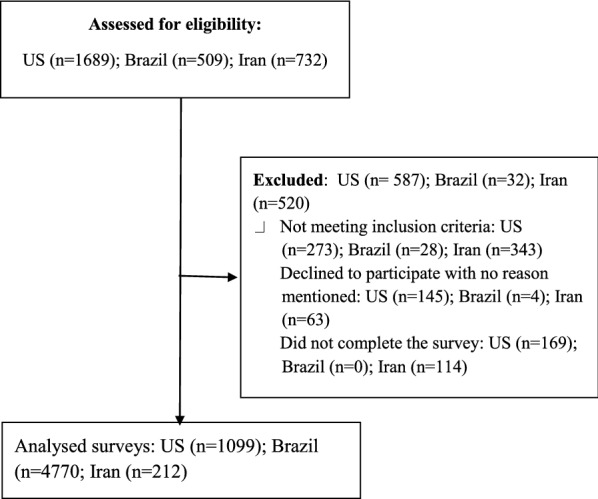
Flow diagram 1: process of study recruitment

In US, of the 1686 individuals who were initially identified, 273 were ineligible, 145 disagreed to participate the study, and 169 did not complete the entire survey, leaving a final sample of 1099 (78% completeness rate). In Brazil, 509 accessed the survey, 4 disagreed with the consent form, 28 were not eligible and 477 completed the survey (94.5% completeness rate). In Iran, 732 individuals completed the screening survey, 343 were ineligible, 63 declined to participate and 114 did not completed the survey, leaving 212 completed survey (54% completeness rate). Please see Flow Diagram 1 in Fig. 1. American and Brazilian participants did not receive an incentive. However, each Iranian participant received a gift card (15,000 Iranian rial) for their time participating at the study.

### Data collection

An interdisciplinary multi-national team of researchers who were knowledgeable about the research field, the culture and language of countries jointly develop the study survey. We developed the survey using web-based tools (in US: QuestionPro; in Brazil: Google Forms, in Iran: Porsline) and translated it to Portuguese and Farsi using translation/back-translation procedure [26]. To prevent biases survey items were randomized and the number of survey items per page was 6. All items included a non-response option such as “not applicable” or “rather not say”, and selection of one response option was enforced. The participants were also able to review and change their answers. The usability and technical functionality of the survey was tested before administrating the survey. We also collected data at the same time periods (April–June 2020) when all three countries where in their COVID-19 peak at that time.

### Study measures

#### Diabetes distress

The Type 1-Diabetes Distress Scale (T1-DDS) including 28 items and 7 subscales (i.e., powerlessness, management distress, hypoglycemia distress, negative social perceptions, eating distress, physician distress, and friend/family distress) was used to assess diabetes distress. T1-DDS utilizes a 6-point Likert scale (1 = not a problem to 6 = serious problem) and had a Cronbach’s alpha of 0.91. Scores of less than 2, 2 to 2.9, and 3 or higher represent cut points for no/mild distress, moderate and high distress, respectively [19].

#### Diabetes burnout

A specific measure of diabetes burnout—Diabetes Burnout Scale (DBS)—was used to assess diabetes burnout. The DBS addresses the main dimensions of diabetes burnout including mental, emotional and physical exhaustion, detachment from illness identity, diabetes self-care, and support systems and loss of control [27]. The DBS is a 12 item scale with a 5-point Likert scale (1 = strongly disagree to 5 = strongly agree). DBS had a Cronbach’s alpha of 0.80. Using a separate item, participants also rated their level of overall burnout (i.e., No, Mild, Moderate, and Severe) based on their own definition of diabetes burnout.

#### Depressive symptoms

To evaluate depressive symptoms, we used the 8-item Patient Health Questionnaire (PHQ-8). PHQ-8 contains 8 items on a four point scale (0 = not at all to 3 = nearly every day) that assess depressive symptoms linked to DSM-V criteria for Major Depressive Disorder (Chronbach alpha = 0.89). Scores of 5, 10, 15, and 20 on the PHQ-8 represent cut points for mild, moderate, moderately severe, and severe depressive symptoms, respectively [28].

#### COVID-19 related questions

We asked specific questions regarding access to diabetes care, diabetes supplies, healthy food, safe places to exercise, and diabetes self-care behaviors prior and during the pandemic. We also asked about diabetes hospitalization and emergency department visits, avoiding diabetes facilities due to fear of becoming infected and following COVID-19 precaution recommendations since the start of the pandemic. Response options were Yes/No for each item.

#### Diabetes outcomes and sociodemographic characteristics

Participants reported their most recent HbA1c within the last 12 months. Participants also reported their daily TiR according to their CGM reading or their perception on the day they completed the survey. Demographic measures included age, gender, education, marital status, residential area, and years of having diabetes.

#### Data analysis

The IP address of the participant device was used to identify potential duplicate entries from the same participant. No two entries from the same IP address were included in data analysis and only completed surveys were analyzed. We used descriptive tests to describe the profile of the sample according to the variables under study. To compare data between countries, when categorical variables were evaluated, we used the Chi-square test and Cramer’s coefficient. We also used the Kruskal–Wallis test, followed by Dunn’s post-hoc test, when numeric variables were evaluated. For comparisons, Bonferroni correction was used for all comparisons involving the three countries.

To define the reference and test classifications, the clinical cutoff points were applied to the scale scores of diabetes distress and depressive symptoms. In the evaluation of the depressive symptoms scale, the moderately severe and severe levels were grouped, due to the low frequency of participants classified as severe in scale. For the distress scale, due to the absence of participants from Iran with classification “No/little” the model was tested for the high level in relation to the others (No/little/moderate). For diabetes burnout we reported mean scores of the total burnout and each burnout dimensions.

The evaluation of factors related to T1-DDS and PHQ-8 was performed through the logistic regression analysis, using the proportional odds model. To assess the factors related to DBS, we used the linear regression analysis. Univariate and multivariate analysis were performed. In the univariate analysis, each variable (factor) was tested independently. Multivariate analysis was performed using the stepwise selection method. We included all variables in the model regardless of the result presented in the univariate analysis. Logistic regression models with country-level Logistic regression models with country-level and the questions in the COVID questionnaire referring to the moment before the start of the pandemic fixed effects were used to evaluate predictors for the scales of distress, burnout, and depressive symptoms. In the logistic regression model, the diabetes distress and depression scales were defined as the dependent variables, considering the “no\little” category as a reference to estimate the chance of presenting a moderate\high level of distress and the “no” category as a reference to estimate the chance of moderate and severe levels of depression. The DBS scale was evaluated considering the ordinal score of the average of the sum of the answers to the questions. Linear regression analysis was also used to assess factors associated with diabetic outcomes. All analyzes were performed using PROC LOGISTIC and PROC REG, SAS System for Windows (Statistical Analysis System), version 9.4. SAS Institute Inc.

## Results

### Demographic descriptive results

Our final sample included 1788 adults with type 1 diabetes from US (n = 1099), Brazil (n = 477), and Iran (n = 212). The majority of participants in all three countries were female. Median age was lowest in Iran (median = 29.00, IRQ = 11.00) and Brazil (median = 28.87, IRQ = 14.00) and median years of having diabetes was lowest in Iran (median = 11.00, IRQ = 7.00) and highest in US (median = 45.00, IRQ = 27.00; median = 25.00, IRQ = 15.00). Brazil and Iran were highest in number of participants living in urban areas (92.24% and 90.57%) compared to US (24.11%). Most participants in Iran (67.45%) and Brazil (59.75%) were single while the majority of respondents from the US were married (61.64%). Iran was also highest in the number of participants having a high school degree (43.87%) compared to Brazil (26.42%) and US (14.23%). Regarding diabetes outcomes, participants in the US sample had the lowest HbA1c (6.97%, 8.5 mmol) and highest TiR (69.64%) compared to Brazil and Iran (Table 1).

**Table 1:** Descriptive statistics—sociodemographic characteristics, diabetes outcomes, and scale results

Sociodemographic	US		Brazil		Iran		P-value	Contingency
	PERC/MEAN	SD	PERC/MEAN	SD	PERC/MEAN	SD		
Gender								
Male	23.69		16.99		24.49		0.0085*	
Female	76.31		83.02		75.51			
Marital status								
With partner	61.64		40.25		32.55		< 0.0001*	0.2296
Without	38.36		59.75		67.45			
Education level								
High school	14.23		26.42		43.87		< 0.0001*	0.2970
Associate	12.23		0.00		6.60			
Bachelor	38.50		46.54		30.66			
Graduate	35.04		27.04		18.87			
Residential area								
Rural	19.00		3.77		9.43		< 0.0001*	0.4730
Urban	24.11		92.24		90.57			
Suburban	56.69		3.98		0.00			

	Median	IQR	Median	IQR	Median	IQR		
Age	45.00	27.00	28.87	11.81	29.00	11.00	< 0.0001†	
Time diagnosis	25.00	15.00	14.00	14.00	11.00	7.00	< 0.0001†	
Diabetes outcomes								
HbA1c	6.97	1.10	7.94	1.75	7.47	1.69	< 0.0001†	
TiR	69.64	18.05	51.95	26.59	51.73	26.22	< 0.0001†	
Scales								
PHQ-8								
No	73.59		47.17		39.15		< 0.0001*	0.2942
Major/Sev major	26.41		52.83		60.85			
T1-DDS								
No/little/moderate	86.60		69.18		42.92		< 0.0001*	0.3437
High	13.40		30.82		57.08			
DBS								
Exhustion	3.22	1.00	3.13	1.16	3.17	1.03	< 0.0001†	
Detachment	1.65	0.64	2.05	1.03	2.72	1.07	< 0.0001†	
Loss of control	2.21	0.92	2.97	0.95	3.29	0.72	< 0.0001†	
DBS-Total	2.31	0.66	2.64	0.85	3.01	0.81	< 0.0001†	
Self-reported overall burnout	3.22	1.00	3.13	1.16	3.17	1.03	< 0.0001†	
Not	28.84		32.29		19.34		< 0.0001†	0.2390
Mild	48.59		36.90		30.66			
Moderate	19.20		30.80		22.17			
Severe	3.37		8.16		27.83			

*Chi-square test

†Kruskal–wallis test

### Perceived challenges prior to and during the pandemic

Table 2 present descriptive results among respondents in the three countries immediately prior to and after the beginning of the pandemic in each country. Significant change (p < 0.05) was noted in response to the pandemic across all three countries across a range of perceived impacts. During the pandemic, participants from all three countries reported dramatic decrease in access to diabetes care (US = 60.1%, Brazil = 51.9%, Iran = 28.3%) compared to prior to the pandemic. Lack of access to diabetes supplies and medication prior to the pandemic increased in all countries during the pandemic (20.8% vs 37.9% for US; 22.1% vs 37.9% for Brazil; 58% vs 67.4% for Iran). At the start of the pandemic, food insecurity increased dramatically in all three countries with the US participants reporting the greatest increase (49.7%), followed by Iran (42%) and Brazil (37.3%). Reported lack of access to safe places to exercise during the pandemic dramatically increased in US (5.9% vs 60.47%), Brazil (7.6% vs 73.3%) and Iran (24.1% vs 81.6%) compared to prior the pandemic. Participants also described perceived changes in their support systems. While 3.6% and 4.7% of American and Brazilians participants lost their support system during the pandemic, there was no change in the number of Iranians with no support system (26.9%).

**Table 2:** COVID-19 related changes questions: prior and during the pandemic

Questions	US		Brazil		Iran	
	Prior	During	Prior	During	Prior	During
Diabetes support system						
Family Friends Providers Support group Others Not	709 (70.8) 101 (10.1) 98 (9.8) 66 (6.6) 26 (2.6) 1 (0.1)	735 (73.4) 91 (9.1) 59 (5.9) 55 (5.5) 24 (2.4) 37 (3.7)	197 (43.9) 12 (2.7) 132 (29.4) 10 (2.2) 15 (3.3) 83 (18.5)	227 (50.6) 16 (3.6) 70 (15.6) 13 (2.9) 19 (4.2) 104 (23.2)	118 (55.7) 8 (3.8) 16 (7.6) 11 (5.2) 2 (0.9) 57 (26.9)	127 (59.9) 9 (4.3) 10 (4.7) 6 (2.8) 3 (1.4) 57 (26.9)
Difficulties accessing supplies or medications						
No Yes	869 (79.2) 228 (20.8)	879 (62.1) 218 (37.9)	350 (77.9) 99 (22.1)	279 (62.1) 170 (37.9)	89 (42.0) 123 (58.0)	69 (32.6) 143 (67.4)
Difficulties accessing healthy food						
No Yes	1071 (97.5) 27 (2.5)	525 (47.8) 573 (52.2)	389 (86.6) 60 (13.4)	201 (44.8) 248 (55.2)	189 (89.2) 23 (10.8)	110 (51.9) 102 (48.1)
Difficulties accessing safe places to excercise						
No Yes	1034 (94.2) 64 (5.8)	664 (60.5) 434 (39.5)	415 (92.4) 34 (7.6)	329 (73.3) 120 (26.7)	161 (75.9) 51 (24.1)	173 (81.6) 39 (18.4)
Difficulties accessing diabetes care						
No Yes	1081 (98.5) 17 (1.5)	422 (38.4) 676 (61.6)	426 (94.9) 23 (5.1)	193 (43.0) 256 (57.0)	192 (90.6) 20 (9.4)	132 (62.3) 80 (37.7)
Changes in self-care behaviours						
No Yes		693 (63.1) 406 (36.9)		272 (60.6) 177 (39.42)		88 (41.5) 124 (58.5)
Diabetes hospitalization/ED visit						
No Yes		1063 (96.7) 36 (3.3)		427 (95.1) 22 (4.9)		149 (70.8) 63 (29.5)
Avoiding diabetes facilities due to fear						
No Yes		573(52.1) 526 (47.9)		134 (29.8) 315 (70.2)		96 (44.8) 117 (55.2)
Following COVID-19 precaution recom						
No Yes		11 (1.0) 1088 (99.0)		9 (2.0) 440 (98.0)		15 (7.1) 197 (92.9)

Since the start of the pandemic, a high percentage of participants in all three countries reported changes in diabetes self-care behaviors (US = 36.9% Brazil = 39.4%, Iran = 58.5%). Diabetes-related hospitalization and ED visits during the pandemic were reported most frequently among Iranian participants (29.3%), followed by Brazil (4.9%) and US (3.3%). Participants in all countries reported following the COVID-19 precaution recommendations ranged from 92.9% in Iran to 99.0% in US. A high number of participants in all three countries also avoided approaching diabetes facilities due to fear of becoming infected by the virus (US = 47.9%; Brazil = 70.2%; Iran = 55.2%).

### Perceived diabetes distress, diabetes burnout, and depressive symptoms during the pandemic

Based on the clinical cut points for the PHQ-8, 26.4% of US participants were classified as having moderately severe or severe depressive symptoms, compared to 52.8% in Brazil and 60.9% in Iran (*p* < 0.0001). Additionally, Iranian sample had the highest prevalence of high distress (57.1%) compared to Brazil (30.8%) and US (13.4%) (*p* < 0.0001). The mean scores of diabetes burnout in different countries varied (Iran = 3.0; Brazil = 2.6; US = 2.3) with statistically significant differences between the three countries (*p* < 0.0001). There was also a significant difference among countries when participants rated their level of burnout (i.e., No, Mild, Moderate, and Severe) based on their overall definition of diabetes burnout (*p* < 0.0001). In Iran 50% reported moderate to severe burnout compared to 30.8% in Brazil and 22.57% in US.

### Predictors and correlates of diabetes distress during the COVID-19 pandemic

Participants with the highest chance of presenting high levels of distress included younger (*p* < 0.0001; OR = 1.031; CI 95% = 1.013–1.048), with a higher levels of HbA1c (*p* < 0.0001; OR: 1.28; CI95% = 1.12–1.46) and lower percentage of TiR (*p* = 0.0002; OR = 1.016; CI 95% = 1.008–1.025). High levels of diabetes distress was also associated with difficulty to access diabetes care (*p* = 0.0032; OR = 1.58; CI 95% = 1.11–2.24) and difficulty to pay for very basic needs (*p* = 0.0243; OR = 1.48; CI 95% = 1.053–2.47). The chance for diabetes distress was higher in those experiencing hospitalization or ED visit (*p* = 0.0004; OR = 3.00; CI 95% = 1.54–5.85) and not following COVID-19 precaution recommendations (*p* = 0.0005; OR = 5.61; CI 95% = 2.14–14.70) (Table 3).

**Table 3:** Predictors of diabetes distress, diabetes burnout and depressive symptoms

Effect^a,b^	P-Value	OR	CI95%
Diabetes distress (dependent variable: T1DDS scale)			
Difficulties to pay for very basic items	0.0182	1.633*	1.078–2.473
Difficulties accessing diabetes care	0.0032	1.583*	1.116–2.245
Diabetes hospitalization or emergency department visit	0.0004	3.008*	1.546–5.856
Not following COVID-19 precaution recommendations	0.0005	5.618*	2.145–14.706
Age^c^	< 0.0001	1.031*	1.013–1.048
HbA1c	< 0.0001	1.285*	1.129–1.461
TiR^c^	0.0002	1.016*	1.008–1.025
Depressive symptoms (dependent variable: PHQ-8 scale)			
Difficulties accessing healthy food	0.0190	1.395**	1.052–1.850
Diabetes self-care benn affected by pandemic	< 0.0001	1.704**	1.278–2.271
Avoiding approaching diabetes facilities due to fear	0.0458	1.335**	1.005–1.774
Age^c^	< 0.0001	1.025**	1.011–1.040
HbA1c	< 0.0001	1.267**	1.122–1.431
TiR^c^	0.0002	1.013**	1.006–1.020
Gender (ref = male)	0.0008	1.835**	1.266–2.660
Marital status (ref = married)	0.0145	1.473**	1.095–1.976
Education (ref = graduate)			
High school	0.0756	1.395**	0.912–2.133
Associate	0.0008	2.738**	1.586–4.726
Bachelor	0.2035	1.149**	0.825–1.600

Effect^a,b^	Beta (CI 95%)	p-value	r-partial	r-model
Diabetes burnout (dependent variable: diabetes burnout scale)				
Difficulties accessing healthy food	0.079 (0.009; 0.149)	0.0280	0.0028	0.3839
Difficulties accessing diabetes care	0.084 (0.010; 0.158)	0.0268	0.0045	0.3772
Diabetes self-care benn affected by pandemic	0.107 (0.033; 0.181)	0.0048	0.0090	0.3727
Avoiding approaching diabetes facilities due to fear	0.078 (0.007; 0.148)	0.0321	0.0024	0.3863
Age^c^	− 0.008 (− 0.011; − 0.005)	< 0.0001	0.0264	0.3637
HbA1c	0.130 (0.101; 0.159)	< 0.0001	0.0476	0.3372
TiR^c^	− 0.011 (− 0.012; − 0.009)	< 0.0001	0.1911	0.2896
Gender (ref = male)	0.107 (0.022; 0.191)	0.0133	0.0039	0.3811

^a^Controlled analysis by the participant's country of origin and the questions in the COVID questionnaire referring to the moment before the start of the pandemic

^b^Variables tested in the model: sociodemographic characteristics, TIR, HbA1C and COVID questionnaire referring to the current moment of the pandemic

^c^Variables inversely associated

*Odds ratio for high distress (reference level: no/little/moderate)

**Odds ratio for moderate severe and severe depressive symptoms (reference level: no/mild)

We also analyzed the predictors of the seven subscales of diabetes distress. Overall, the results were consistent with all the predictors of the total diabetes distress score (i.e., age, HbA1c, difficulties accessing diabetes care). However, the analysis revealed the additional associations between gender and Powerlessness distress, education and hospitalization with Hypoglycemia distress, Time of diagnosis with Eating distress, residential area with Physician distress, healthy food and safe places to exercise with Family/Friend distress subscales (all *p* < 0.05) (Additional file 1: Table S1).

### Predictors and correlated of diabetes burnout during the COVID-19 pandemic

Participants who had the highest levels of diabetes burnout included women (*p* = 0.0133; β = 0.17 [− 0.02; − 0.19]; *r*^*2*^ = 0.38), younger (*p* < 0.0001; β = − 0.008 [− 0.011; − 0.005]; *r*^*2*^ = 0.03), with a higher HbA1c (*p* < 0.0001; β = 0.13 [0.10; 0.15]; *r*^*2*^ = 0.04) and lower TiR (*p* < 0.0001; β = − 0.01 [− 0.01; − 0.009]; *r*^*2*^ = 0.28). The level of diabetes burnout was higher for participants experiencing difficulties to access to healthy food (*p* = 0.0280; β = 0.079 [0.00; 0.14]; *r*^*2*^ = 0.38), changes in diabetes self-care behaviors (*p* = 0.0048; β = 0.107 [0.03; 0.18]; *r*^*2*^ = 0.37), and difficulty accessing diabetes care (p = 0.0268; β = 0.084 [0.01; 0.15]; r2 = 0.37) (Table 3).

### Predictors and correlated of depressive symptoms during the COVID-19 pandemic

Participants who had the highest chance of depressive symptoms included women (*p* = 0.0008; OR = 1.83; CI95% = 1.26–2.66), younger (*p* < 0.0001; OR = 1.025; CI95% = 1.011–1.040), single (*p* = 0.0145; OR = 1.47; CI95% = 1.09–1.97) with associate education level (*p* = 0.0032; OR = 2.738; CI95% = 1.586–4.726), higher HbA1c (*p* < 0.000; OR = 1.26; CI95% = 1.12–1.43) and lower TiR (*p* = 0.0002; OR = 1.013; CI95% = 1.006–1.020). The chance of depressive symptoms was greater for participants who experienced difficulties to access to healthy food (*p* = 0.0190; OR = 1.39; CI95% = 1.05–1.85), changes in diabetes self-care behaviors (*p* < 0.0001; OR = 1.70; CI95% = 1.27–2.27), avoiding approaching diabetes facilities due to fear (*p* = 0.0458; OR = 1.33; CI95% = 1.05–1.77) (Table 3).

### Predictors and correlates of diabetes outcomes during the COVID-19 pandemic

Participants with higher levels of HbA1c and lower levels of TiR included younger, single, with lower educational level. During the pandemic, limited access to healthy food was associated with an increase of 0.29-point in HbA1c (*p* = 0.04; *b* = 0.29 [0.01; 0.58]; *r*^*2*^ = 0.0023). Similarly, lack of access to diabetes care was associated with a 6.75 decrease in TiR (*p* = 0.03; *b* = − 6.75 [− 13.02; − 0.48]; *r*^*2*^ = 0.0025). (Table 4).

**Table 4:** Predictor of diabetes outcomes

	Beta (CI 95%)	P-value	r^2^	r-adj
Dependent: HbA1c				
Lack of access to healthy food during the pandemic	0.29 (0.01; 0.58)	0.0437	0.0023	0.1620
Education (ref graduate):				
High school	0.51 (0.34; 0.68)	< 0.0001	0.0257	0.1239
Associate	0.44 (0.20; 0.68)	0.0003	0.0092	0.1498
Marital status (ref married):				
Single	0.22 (0.08; 0.36)	0.0017	0.0056	0.1554
Age	− 0.01 (− 0.01; 0.00)	0.0001	0.0167	0.1406
Dependent: TiR				
Lack of access to diabetes care during the pandemic	− 6.75 (− 13.02; − 0.48)	0.0349	0.0025	0.1755
Education (ref graduate):				
High school	− 6.84 (− 9.69; − 3.99)	< 0.0001	0.0171	0.1522
Associate	− 5.52 (− 9.48; − 1.56)	0.0064	0.0049	0.1687
Marital status (ref married) Single	− 2.32 (− 4.60; − 0.03)	0.0471	0.0022	0.1777
Age^a^	− 0.15 (− 0.24; − 0.07)	0.0003	0.0116	0.1638

^a^Variables inversely associated

## Discussion

### Perceived challenges prior to and during the pandemic

The US, Brazil, and Iran are different in a variety of ways and the impact of the pandemic and crisis management and policy responses within each country are also asymmetric. Although, our findings suggest all countries experienced a dramatic decrease in access to diabetes care, diabetes supplies and medication, healthy food, and safe places to exercise. Our findings are similar to other studies highlighting resource disparities as common challenges facing individuals with diabetes in emergency circumstances [29, 30]. These countries have also a government stringency index of approximately 71% (a composite measure based on nine COVID-19 response indicators) [3]. However, differences in reported challenges by participants in these countries may suggest that the government initiatives and innovations in different countries have not been sufficient to eliminate the growing COVID-19 disparities among individuals with diabetes [31–33].

Avoiding diabetes facilities due to fear of being infected was extremely high in all three countries. Fear of becoming infected as a barrier to access to diabetes care is reported in other studies [34, 37, 38]. The mass media coverage of the pandemic, the alarming mortality and incidence rates of the COVID-19, social isolation, the risk of potentially being more vulnerable to the virus, changes in routine diabetes care are anticipated to intensify fear among individuals with high-risk conditions including diabetes [37, 39, 40].

### Perceived diabetes distress, diabetes burnout, and depressive symptoms during the pandemic

A high number of participants in all countries reported high levels of diabetes distress, diabetes burnout, and depressive symptoms; however, it was higher in Brazil and Iran. Evidence suggest that under normal circumstances around 40% of individuals with diabetes experience moderate to severe diabetes distress and depressive symptoms [36] and diabetes burnout [21]. The results of diabetes distress and diabetes burnout in our US sample are comparable with other studies at non pandemic circumstances [23, 27]. However, the participants reported a higher prevalence of severe depressive symptoms compared to previous studies [42]. The lower prevalence of diabetes distress and diabetes burnout in the US sample may be explained in part by including more participants from US rural areas, possibly experiencing less distress and burnout.

Our sample from Brazil and Iran also reported higher prevalence of diabetes distress (30.8%, 57.1%) and depressive symptoms (52.8%, 69.9%) in compared to other studies in individuals with diabetes during non-pandemic situations [43, 44]. Although, these results are expectable during pandemic circumstances, higher levels of distress and depression has been reported in general population of Brazil and Iran [45, 46]. These can be associated to social, financial, and political situations of these countries even before the pandemic. The Brazdiab, a large nationwide multicenter diabetes study in Brazil in 2013 showed that approximately 68% of individuals struggling to manage diabetes care cost. They may reuse disposable supplies, reduce doses of medication, or perform SMBG less often than recommended [47]. Prior to the pandemic, suboptimal diabetes outcomes, shortage of diabetes care providers, lack of access to diabetes care, high financial burden of diabetes care (i.e., cost of medicine and strips), and lack of diabetes education have been reported as existing disparities in Brazil and Iran [34–36, 38, 48]. The pre-existing disparities in Brazil and Iran and share comparable combined with the psychosocial consequences of the pandemic and financial crisis in these countries may add to the burden of psychosocial problems of individuals with diabetes in these countries.

### Associations of perceived COVID-19 challenges, demographic, and outcome variables

We found that individuals experiencing difficulties to pay for very basic needs, hospitalization, limited access to healthy food and diabetes care, changes in self-care routines, fear of becoming infected are most likely to present higher levels of psychosocial issues. Like other studies, vulnerable groups disproportionally experience the burden of the pandemic that may pose more risk for adverse psychosocial consequences [49]. We also found that individuals with difficulty to access healthy food and diabetes care during the pandemic were more vulnerable to experience suboptimal glycemic control. These are similar to other studies identified access to healthy food and medical services as possible factors responsible for adverse glycemic control during the pandemic [50, 51].

Our study showed that female, younger, single participants with lower educational levels, Higher HbA1c, and lower TiR are most likely to present lower levels of psychosocial well-being. This is consistent with other evidence, suggesting the associations between demographic variables, suboptimal psychosocial well-being and poor glycemic control [52]. Significance of age, gender, education, and marital status suggest that individuals with different demographic characteristics respond differently to the pandemic and these populations are more vulnerable and should require individualized psychosocial care.

### Study limitation

Participants’ responses can be affected by the specific cultural, context and socio-determinants of health in each country. Therefore, the study may suffer from measurement, sampling, and data collection bias. Differences in the ability to participate between countries (i.e., internet access) may impact the results. The participants also may not be representative within their countries, so comparisons between countries and interpretation of the results should be taken with caution. Unequal samples size may impact the regression analysis as the US had the largest sample size. However, the sample sizes on the other two countries are not small in and of themselves. Providing incentives to Iranian sample may cause selection bias. It is also likely that those experiencing poor psychosocial well-being participate less in diabetes research studies and therefore the sample may not represent individuals with diabetes experiencing different levels of diabetes distress, diabetes burnout, and depressive symptoms. Another limitation is that the study outcome measures and COVID-19 related questions were based on self-reports. Cultural desirability and memory recall can introduce unwanted and systematic errors. The majority of participants were female. We did not collected data on diabetes hospitalization/ED visit before the pandemic and more clearly note that limitation. In addition not all participants had CGM. Therefore, TiR was a perception of participants without CGM during the day.

## Conclusions

The study results may suggest that regardless of significant contextual, social, and financial differences among countries, individuals with type 1 diabetes are experiencing suboptimal psychosocial well-being during the pandemic and face similar challenges related to the COVID-19 pandemic. Increasing access to diabetes care, supplies, healthy food, and safe places to exercise requires a collaborative approach among various sectors globally. The need for flexibility and adaptability of diabetes care, education, and social support is inevitable. Diabetes practices and healthcare providers should reassess the specific needs of their populations and coordinate and prioritize available resources to address them. Providing high quality digital diabetes care and support, distributing adequate medications and diabetes supplies, and HbA1c home based kit are necessary. Psychosocial support (i.e. frequent text messaging, phone call) should be evolved and adapted to the needs of individuals with type 1 diabetes affected by COVID-19 to maintain both physical and psychosocial well-being and improve diabetes outcomes. Modifiable variables (i.e., access to healthy food and diabetes care) should also be priorities of governments while navigating to mitigate the impact of the pandemic on individuals with diabetes.

We call for systematic and periodic psychosocial assessment for all individuals with diabetes and ask for development and implementations of feasible and effective psychosocial interventions for this vulnerable population particularly during the pandemic.

## Supplementary Information

**Additional file 1:** **Table S1.** Predictors of diabetes distress subscales (multivariate model).

〹

## Data Availability

All data generated or analyzed during this study are included in this published article.
