# Supplementary material for: Cross-national comparison of psychosocial well-being and diabetes outcomes in adults with type 1 diabetes during the COVID-19 pandemic in US, Brazil, and Iran
Source: Diabetol Metab Syndr. 2021 Jun 11;13:63. doi: 10.1186/s13098-021-00681-0 (PMC8193175; doi:10.1186/s13098-021-00681-0)
Supplement: Supplementary file 1 — Additional file 1: Table S1. Predictors of diabetes distress subscales (multivariate model). [file 13098_2021_681_MOESM1_ESM.docx]

Table S1: Predictors of diabetes distress subscales (multivariate model)

| Effect ^1,2^^[[1]](#footnote-1)^ | P-Value | OR^*^ | CI95% |
| --- | --- | --- | --- |
| **T1-DDS Powerlessness** | | | |
| Not following COVID-19 precausion recommendations  Distruption to diabetes self-care rutine  Avoiding diabetes facilities due to fear  Age^†^  HbA1c  TiR^†^  Gender (ref=male) | 0.0030  <.0001  0.0033  <.0001  <.0001  0.0006  0.0400 | 5.051  1.764  1.454  1.034  1.283  1.012  1.410 | 1.761-14.493  1.353-2.299  1.118-1.891  1.022-1.046  1.140-1.444  1.005-1.019  1.015-1.961 |
| **T1-DDS Management** | | | |
| Difficulties accessing safe place to excercise  Not following COVID-19 precausion recommendations  Distruption to diabetes self-care rutine  Age^†^  HbA1c  TiR^†^ | 0.0117  0.0369  0.0101  <.0001  <.0001  <.0001 | 1.692  2.710  1.705  1.043  1.535  1.027 | 1.135-2.519  1.047-7.874  1.170-2.484  1.024-1.063  1.328-1.775  1.018-1.035 |
| **T1-DDS Hypoglycemia** | | | |
| Difficulties accessing healthy food  Difficulties accessing diabetes care  Diabetes hospitalization or emergency department visit  TiR^†^  Gender (ref=male)  Education (ref=Graduate)  High school  Associate  Bachelor | 0.0143  0.0016  <.0001  0.0002  0.0245  0.0059 | 1.398  1.473  3.414  1.010  1.515  1.876  1.542  1.055 | 1.054-1.854  1.105-1.963  1.823-6.395  1.004-1.016  1.054-2.183  1.252-2.809  0.885-2.685  0.758-1.467 |
| **T1-DDS Negative Social Perception** | | | |
| Difficulties accessing diabetes care  Not following COVID-19 precausion recommendations  Age  TiR | 0.0012  0.0014  <.0001  0.0010 | 1.802  4.132  1.034  1.012 | 1.298-2.501  1.664-10.309  1.018-1.050  1.005-1.018 |
| **T1-DDS Eating** | | | |
| Difficulties accessing diabetes care  Age^†^  Time diag^†^  HbA1c  TiR^†^  Gender (ref=male) | 0.0084  <.0001  0.0381  <.0001  0.0002  0.0160 | 1.422  1.020  1.013  1.275  1.013  1.553 | 1.090-1.856  1.007-1.034  1.001-1.026  1.135-1.432  1.006-1.020  1.114-2.165 |
| **T1-DDS Physician** | | | |
| Difficulties accessing healthy food  Difficulties accessing diabetes care  Not following COVID-19 precausion recommendations  Age^†^  HbA1c  TiR^†^  Residential area (ref=suburban)  Urban  Rural | 0.0005  0.0198  0.0428  0.0003  <.0001  0.0389  0.0408 | 1.716  1.591  2.778  1.027  1.190  1.009  1.974  1.772 | 1.195-2.466  1.104-2.294  1.020-7.578  1.009-1.044  1.048-1.350  1.001-1.018  1.092-3.566  1.029-3.050 |
| **T1-DDS Friend/Family** | | | |
| Difficulties accessing healthy food  Difficulties accessing safe place to excercise  Difficulties accessing diabetes care  Age^†^  TiR^†^  Education (ref=graduate)  High school  Associate  Bachelor | 0.0012  0.0324  0.0107  <.0001  0.0114  0.0003 | 1.629  1.535  1.532  1.036  1.009  2.378  1.830  1.143 | 1.152-2.302  1.035-2.278  1.078-2.176  1.019-1.055  1.002-1.016  1.454-3.892  0.891-3.759  0.739-1.768 |

1. ^Controlled analysis by the participant's country of origin and the questions in the COVID questionnaire referring to the moment before the start of the pandemic.^

   2 ^Variables tested in the model: sociodemographic characteristics, TiR, HbA1c and COVID questionnaire referring to the current moment of the pandemic.^

   ^*^ ^Odds ratio for high distress (reference level: no/little/moderate).^

   ^**^ ^Odds ratio for moderate severe and severe depressive symptoms (reference level: no/mild).^

   ^† Variables inversely associated.^  [↑](#footnote-ref-1)
